# Machine learning and complex biological data

**DOI:** 10.1186/s13059-019-1689-0

**Published:** 2019-04-16

**Authors:** Chunming Xu, Scott A. Jackson

**Affiliations:** 1 Center for Applied Genetic Technologies, Institute for Plant Breeding, Genetics and Genomics, The University of Georgia, 111 Riverbend Rd, Athens, GA 30602 USA; 2 Key Laboratory of Molecular Epigenetics of the Ministry of Education (MOE), Northeast Normal University, Changchun, 130024 China

## Abstract

Machine learning has demonstrated potential in analyzing large, complex biological data. In practice, however, biological information is required in addition to machine learning for successful application.

## The revolution of biological techniques and demands for new data mining methods

**Fig. 1:**
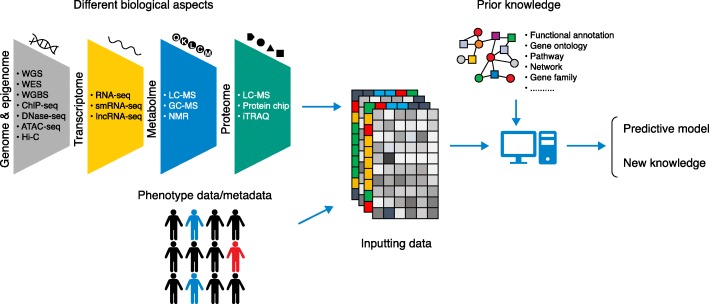
Machine learning using complex biological data. High-throughput data generation techniques for different biological aspects are shown (*left*). *ATAC-seq* assay for transposase-accessible chromatin using sequencing, *ChIP-seq* chromatin immunoprecipitation sequencing, *DNase-seq* DNase I hypersensitive sites sequencing, *GC-MS* gas chromatography-mass spectrometry, *LC-MS* liquid chromatography–mass spectrometry, *lncRNA-seq* long non-coding RNA sequencing, *NMR* nuclear magnetic resonance, *RNA-seq* RNA sequencing, *smRNA-seq* small RNA sequencing, *WES* whole exome sequencing, *WGBS* whole-genome bisulfite sequencing, *WGS* whole genome sequencing, *Hi-C* chromatin conformation capture combined with deep sequencing, *iTRAQ* isobaric tags for relative and absolute quantification

In order to more completely understand complex biological phenomena, such as many human diseases or quantitative traits in animals/plants, massive amounts and multiple types of ‘big’ data are generated from complicated studies. In the not so distant past, data generation was the bottleneck, now it is data mining, or extracting useful biological insights from large, complicated datasets. In the past decade, technological advances in data generation have advanced studies of complex biological phenomena. In particular, next generation sequencing (NGS) technologies have allowed researchers to screen changes at varying biological scales, such as genome-wide genetic variation, gene expression and small RNA abundance, epigenetic modifications, protein binding motifs, and chromosome conformation in a high-throughput and cost-efficient manner (Fig. 1). The explosion of data, especially omics data (Fig. 1), challenges the long-standing methodologies for data analysis.

Biological systems are complex. Most large-scale studies focus only on one specific aspect of the biological system; for example, genome-wide association studies (GWAS) focus on genetic variants associated with measured phenotypes. However, complex biological phenomena can involve many biological aspects, both intrinsic and extrinsic (Fig. 1), and, thus, cannot be fully explained using a single data type. For this reason, the integrated analysis of different data types has been attracting more attention. Integration of different data types should, in theory, lead to a more holistic understanding of complex biological phenomena, but this is difficult due to the challenges of heterogeneous data and the implicitly noisy nature of biological data [1]. Another challenge is data dimensionality: omics data are high resolution, or stated another way, highly dimensional. In biological studies, the number of samples is often limited and much fewer than the number of variables due to costs or available sources (e.g., cancer samples, plant/animal replicates); this is also referred to as the ‘curse of dimensionality’, which may lead to data sparsity, multicollinearity, multiple testing, and overfitting [2].

## Machine learning versus statistics

The boundary between machine learning and statistics is fuzzy. Some methods are common to both domains and either can be used for prediction and inference. However, machine learning and statistics have different foci, prediction or inference [3]. In general, classic statistical methods rely on assumptions about the data-generating systems. Statistics can provide explicit inferences through fitting a specified probability model when enough data are collected from well-designed studies. Machine learning is concerned with the question of creation and application of algorithms that improve with experience. Many machine learning methods can derive models for pattern recognition, classification, and prediction from existing data and do not rely on stringent assumptions about the data-generating systems, which makes them more effective in some complicated applications, as further described below, but less effective in producing explicit models with biological significance, in some cases [3].

## The applications of machine learning in biology

There are two primary types of machine learning methods: supervised learning and unsupervised learning. Supervised learning algorithms learn the relationship between a set of input variables and a designated dependent variable or labels from training instances and can subsequently be used to predict the outcomes of new instances. Many sophisticated machine learning methods are supervised, e.g., decision tree, support vector machine, and neural network. Unsupervised learning algorithms infer patterns from data without a dependent variable or known labels. Cluster and principle component analysis are two popular unsupervised learning methods used to find patterns in high dimensionality data such as omics data. Deep learning is a subtype of machine learning originally inspired by neuroscience, essentially describing a class of large neural networks. Deep learning has been applied in many fields, largely driven by the massive increases in both computational power and big data. Deep learning can be both supervised and unsupervised, has revolutionized fields such as image recognition, and shows promise for applications in genomics, medicine, and healthcare.

Machine learning has been used broadly in biological studies for prediction and discovery. With the increasing availability of more and different types of omics data, the application of machine learning methods, especially deep learning approaches, has become more frequent. One area of opportunity for machine learning approaches is in the prediction of genomic features, particularly those that are hard to predict using current approaches such as regulatory regions. Machine learning has been used to predict the sequence specificities of DNA- and RNA-binding proteins, enhancers, and other regulatory regions [4, 5] on data generated by one or multiple types of omics approach, such as DNase I hypersensitive sites (DNase-seq), formaldehyde-assisted isolation of regulatory elements with sequencing (FAIRE-seq), assay for transposase-accessible chromatin using sequencing (ATAC-seq), and self-transcribing active regulatory region sequencing (STARR-seq). Machine learning can be used to build models to predict regulatory elements and non-coding variant effects de novo from a DNA sequence [5] that can then be tested/validated for their contribution to gene regulation and ultimately to observable traits/pathologies.

In addition to the prediction of regulatory regions, recently, supervised learning showed considerable potential for solving population and evolutionary genetics questions, such as the identification of regions under purifying selection or selective sweeps, as well as more complicated spatiotemporal questions (reviewed in [6]). Up to now, machine learning approaches have also been used to predict transcript abundance [7], imputation of missing SNPs and DNA methylation states [8, 9],variant calling [10], disease diagnosis/classification, and many different biological questions using datasets from different biological aspects such as genomes, epigenomes, transcriptomes, and metabolomes.

## Challenges and future outlooks

**Fig. 2:**
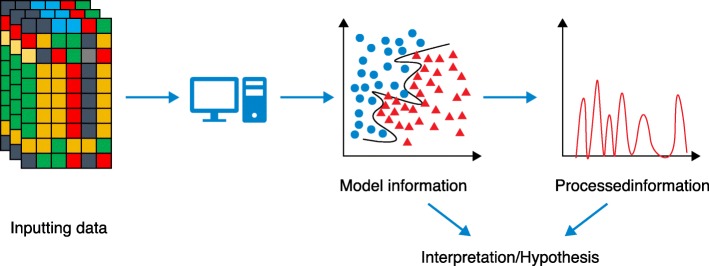
Interpretation of machine learning model. Model information may be interpreted directly or be further processed for better understanding

The massive and rapid advancements in both biological data generation and machine learning methodologies are promising for the analysis and discovery from complex biological data. However, there are several hurdles. Firstly, interpretation of models derived from some sophisticated machine learning approaches such as deep learning can be difficult if not impossible. In many cases, researchers are more interested in the biological meaning of the predictive model than the predictive accuracy of the model and the ‘black box’ nature of the model can inhibit interpretation. The information from the model may need further processing and should be carefully interpreted with corresponding biological knowledge (Fig. 2).

Although several methods have been developed for interpreting and understanding complicated models, such as perturbation-based methods and gradient-based methods for the interpretation of convolutional neural networks (CNNs), the interpretation of many complicated cases may yet be challenging and currently out of reach. Joint analysis of multiple biological data types has the potential to further our understanding of complex biological phenomena; however, data integration is challenging due to the heterogeneity of different data types. For example, an expression profile is a vector of real values and the length of vector is equal to the number of genes in the genome, while the genetic variants are categorial and of different vector length. Various strategies for data integration have been used in different studies [1, 4] but best practices about which data types can be integrated and how to integrate data are still needed.

Another challenge is the curse of dimensionality. Problems such as sparsity, multicollinearity, and overfitting are difficult to avoid in high-resolution studies such as in omics datasets, although the larger sample size and modern machine learning methods can partially mitigate these problems [2]. To increase the number of samples it may be necessary to combine data from multiple sources, which may be feasible for qualitative data like single-nucleotide polymorphisms (SNPs) but can be hard for quantitative data such as gene expression data due to the many ‘hidden’ effects such as variation in developing stages or batch effects from experimental methodologies that can confound analyses. It is still an open question how to normalize data from different sources and additional work on data production, sharing, and processing will be necessary.

Although improved machine learning methods and the increasing number of available samples show great promise to increase our understanding of complex biological phenomena, building proper machine-learning models can still be challenging due to hidden biological factors such as population structure among samples or evolutionary relationship among genes. Biological datasets should be carefully curated to remove confounders. Without properly accounting for such factors, the models can be overfit, leading to false-positive discovery. To build proper models, the biological and technical factors specific to the modeling scenario need to be taken into account. For example, biological data are often imbalanced, such as the case in some diseases or traits that occur only in a small fraction of a population. It is usually more meaningful to access metrics like precision and recall for the non-major class rather than simple accuracy to evaluate model performance for imbalanced classes in the data.

Traditional statistical approaches still dominate the biological research field, even for large omics data analyses. However, the flood of omics data across scales, cells to tissues to organisms to ecosystems, and types, genotyping, resequencing, RNA-seq, bisulfite sequencing (BS-seq), etc., and new more powerful machine learning methods, hold great promise to provide biological insights from the large and often heterogeneous data. Different machine learning methods may correspond to underlying assumptions about data; for example, two popular deep learning methods, convolutional neural network (CNN) and recurrent neural network (RNN), were designed for different types of data. No single computational approach or rule is suitable for all biological questions. Rather, each complex biological question will require specific machine learning approaches, e.g., support vector machine, random forest, and deep neural network, and combinations of disciplines, e.g. computer science, statistics, physics, engineering, and biology. We predict that researchers who are capable of applying machine learning to complex biological data will be increasingly in demand in the future.
